# Supplementary material for: A comparison of drying methods on the quality for bryophyte molecular specimens collected in the field
Source: PLoS One. 2022 Nov 23;17(11):e0277778. doi: 10.1371/journal.pone.0277778 (PMC9683613; doi:10.1371/journal.pone.0277778)
Supplement: S1 Table — 150, 150°C hot-air drying; 80, 80°C hot-air drying; 40, 40°C hot-air drying; N, natural drying; S, silica gel drying; F, fresh sample; a,b,c,d The superscript of same letters indicate that there is no statistically significant difference (P>0.05), the superscript of different letters indicate that there is a statistically significant difference (P<0.05). (DOCX) [file pone.0277778.s001.docx]

**S1 Table Comparison of OD 260/230 values of the four bryophytes’ DNA after different drying treatments**

| **Treatments** | ***C. schmidii*** | ***P. commune*** | ***H. calcicola*** | ***M. polymorpha*** |
| --- | --- | --- | --- | --- |
| 150 | 1.010(0.12)^b^ | 1.650(0.17)^a^ | 1.680(0.25)^ab^ | 2.140(0.10)^b^ |
| 80 | 1.080(0.06)^b^ | 1.675(0.12)^a^ | 1.850(0.24)^a^ | 2.200(0.06)^a^ |
| 40 | 1.230(0.15)^b^ | 1.325(0.20)^b^ | 1.670(0.19)^ab^ | 2.240(0.09)^a^ |
| N | 1.030(0.19)^b^ | 1.400(0.22)^b^ | 1.650(0.17)^ab^ | 2.210(0.08)^a^ |
| S | 1.160(0.31)^b^ | 1.230(0.21)^c^ | 1.510(0.22)^b^ | 2.130(0.15)^b^ |
| F | 1.360(0.25)^a^ | 1.670(0.20)^a^ | 1.620(0.27)^ab^ | 2.090(0.07)^b^ |

150, 150°C hot-air drying; 80, 80°C hot-air drying; 40, 40°C hot-air drying; N, natural drying; S, silica gel drying; F, fresh sample; ^a,b,c,d^The superscript of same letters indicate that there is no statistically significant difference (P>0.05), the superscript of different letters indicate that there is a statistically significant difference (P<0.05).
