# Supplementary material for: A comparison of drying methods on the quality for bryophyte molecular specimens collected in the field
Source: PLoS One. 2022 Nov 23;17(11):e0277778. doi: 10.1371/journal.pone.0277778 (PMC9683613; doi:10.1371/journal.pone.0277778)
Supplement: S2 Table — 150, 150°C hot-air drying; 80, 80°C hot-air drying; 40, 40°C hot-air drying; N, natural drying; S, silica gel drying; F, fresh sample; DNA-N means total DNA concentration form Nanodrop 2000 micro-spectrophotometer and DNA-G means long fragment DNA from agarose gel electrophoretogram. a,b,c,d The superscripts of the same letters indicate that there is no statistically significant difference (P>0.05). The superscripts of the different letters indicate that there is a statistically significant difference (P<0.05) −1. (DOCX) [file pone.0277778.s002.docx]

**S2 Table Comparisons of extract DNA concentrations of the four bryophytes after different drying treatments**

| **Treat-ments** | **DNA-N (ng·μl^−1^)** | | | | **DNA-G (ng·μl^−1^)** | | | |
| --- | --- | --- | --- | --- | --- | --- | --- | --- |
|  | *C. schmidii* | *P. commune* | *H. calcicola* | *M. polymorpha* | *C. schmidii* | *P. commune* | *H. calcicola* | *M. polymorpha* |
| **150** | 24.9(8.6)^b^ | 43.5(16.49)^b^ | 8.3(2.1)^d^ | 155.9(62.4)^a^ | 0.19(0.63)^b^ | 0.59(0.65)^c^ | 0.00(0.00)^b^ | 0.16 (0.25)^b^ |
| **80** | 27.1(4.9)^b^ | 62.2(21.73)^a^ | 11.5(3.5)^cd^ | 152.9(49.1 )^a^ | 1.80(2.32)^b^ | 1.37(1.24)^b^ | 0.00(0.00)^b^ | 8.70(3.11)^a^ |
| **40** | 22.1(7.7)^b^ | 39.15(21.34)^b^ | 15.1(4.2)^bc^ | 149.7(43.4)^ab^ | 4.76(4.45)^a^ | 0.00(0.93)^c^ | 0.00(0.00)^b^ | 7.93(3.31)^a^ |
| **N** | 18.7(6.6 )^b^ | 33.65(13.48)^bc^ | 16.7(4.0)^b^ | 109.1(34.2)^ab^ | 0.93(0.60)^b^ | 0.00(0.00)^c^ | 0.00(0.00)^b^ | 5.71(2.61)^a^ |
| **S** | 19(8.4)^b^ | 26.05(9.01)^c^ | 14.9(3.0)^bc^ | 109.4(58.6)^ab^ | 2.00(2.02)^b^ | 0.00(0.76)^c^ | 0.00(0.00)^b^ | 6.62(3.87)^a^ |
| **F** | 34.3(12.6)^a^ | 66.6(22.26)^a^ | 22.2(9.5)^a^ | 104.8(32.0)^b^ | 2.02(1.87)^b^ | 3.20(2.74)^a^ | 0.61(3.94)^a^ | 5.62(3.30)^a^ |

150, 150°C hot-air drying; 80, 80°C hot-air drying; 40, 40°C hot-air drying; N, natural drying; S, silica gel drying; F, fresh sample; DNA-N means total DNA concentration form Nanodrop 2000 micro-spectrophotometer and DNA-G means long fragment DNA from agarose gel electrophoretogram. ^a,b,c,d^The superscripts of the same letters indicate that there is no statistically significant difference (P>0.05). The superscripts of the different letters indicate that there is a statistically significant difference (P<0.05) ^−1^.
