# Supplementary figures and images for: A comparison of drying methods on the quality for bryophyte molecular specimens collected in the field
Source: PLoS One. 2022 Nov 23;17(11):e0277778. doi: 10.1371/journal.pone.0277778 (PMC9683613; doi:10.1371/journal.pone.0277778)

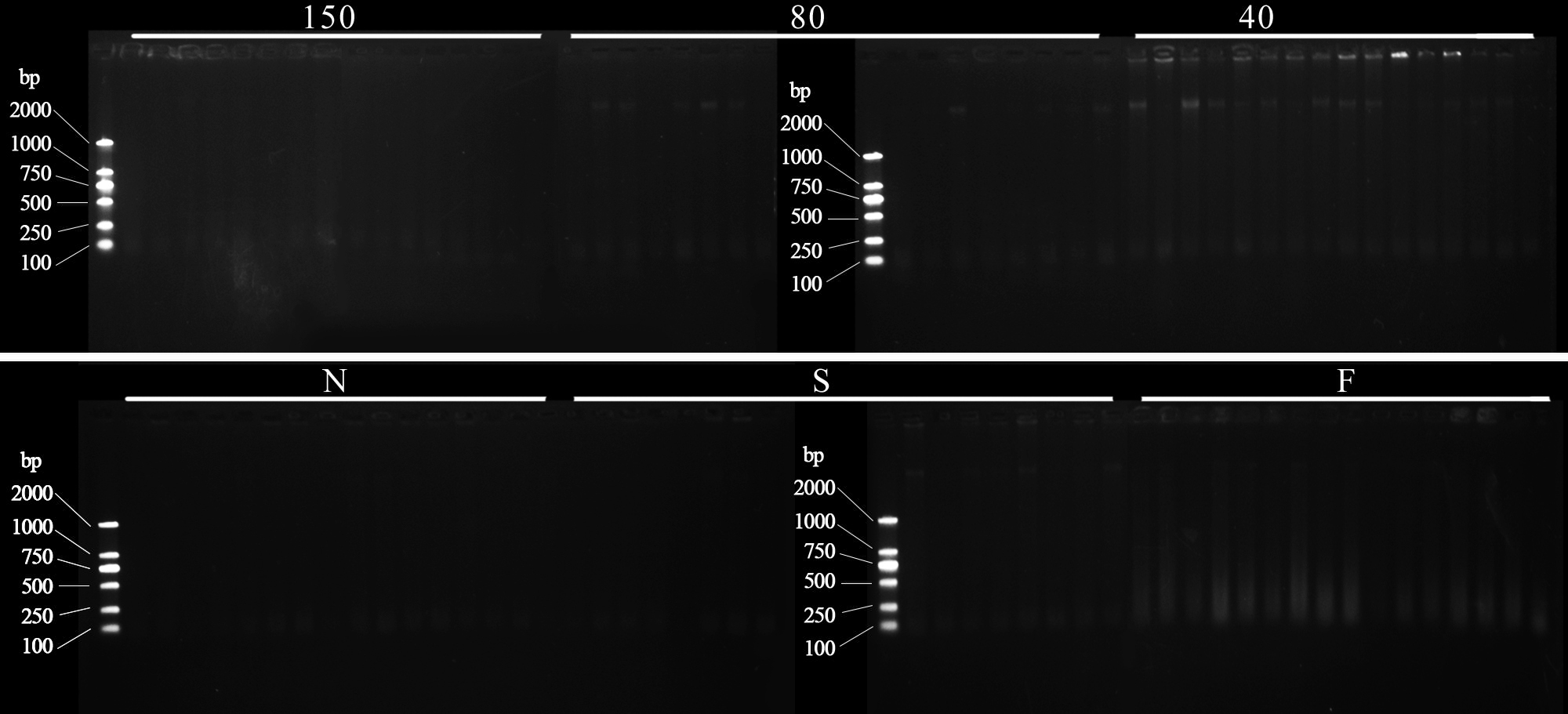

Supplement: S1 Fig — Note, 150, 150°C hot-air drying; 80, 80°C hot-air drying; 40, 40°C hot-air drying; N, natural drying; S, silica gel drying. (TIF) [file pone.0277778.s003.tif]

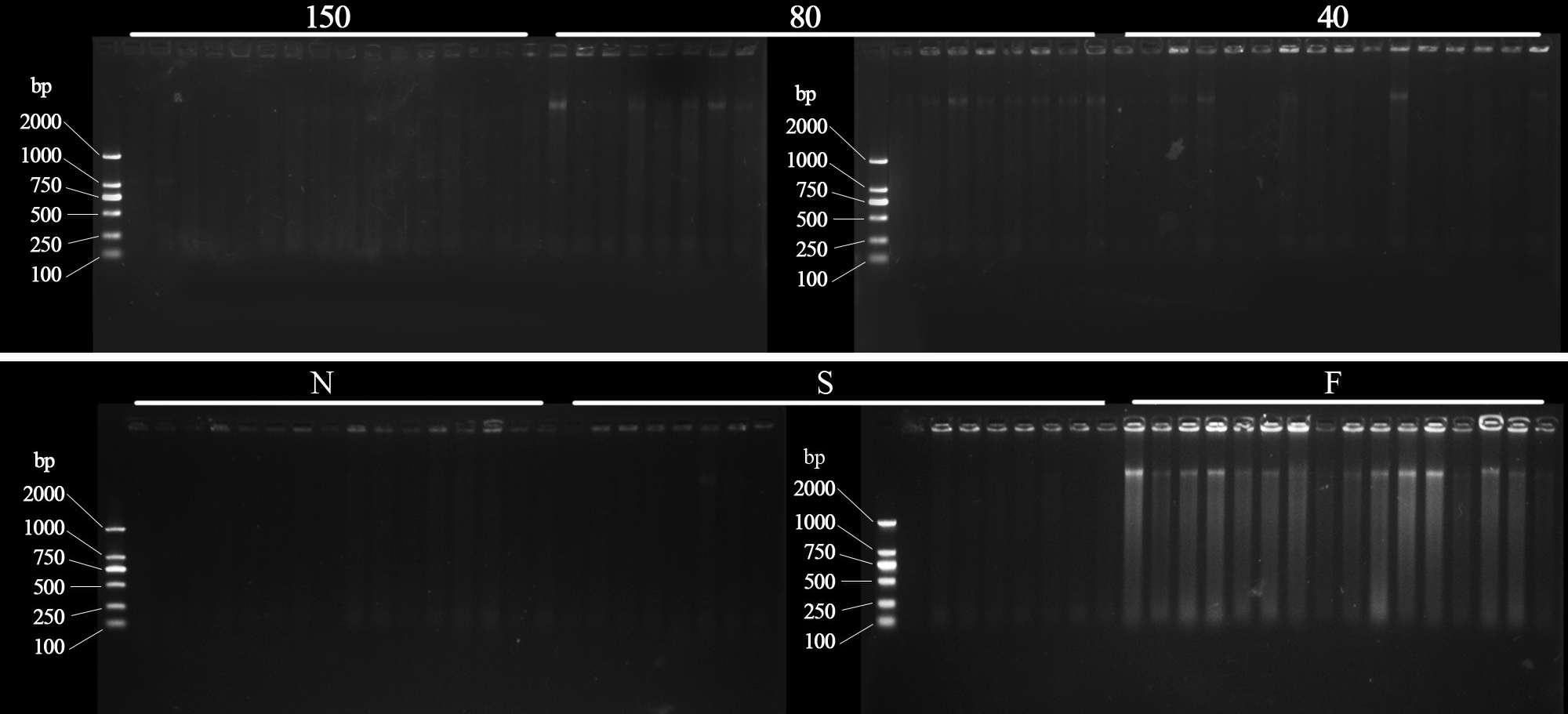

Supplement: S2 Fig — Note, 150, 150°C hot-air drying; 80, 80°C hot-air drying; 40, 40°C hot-air drying; N, natural drying; S, silica gel drying. (TIF) [file pone.0277778.s004.tif]

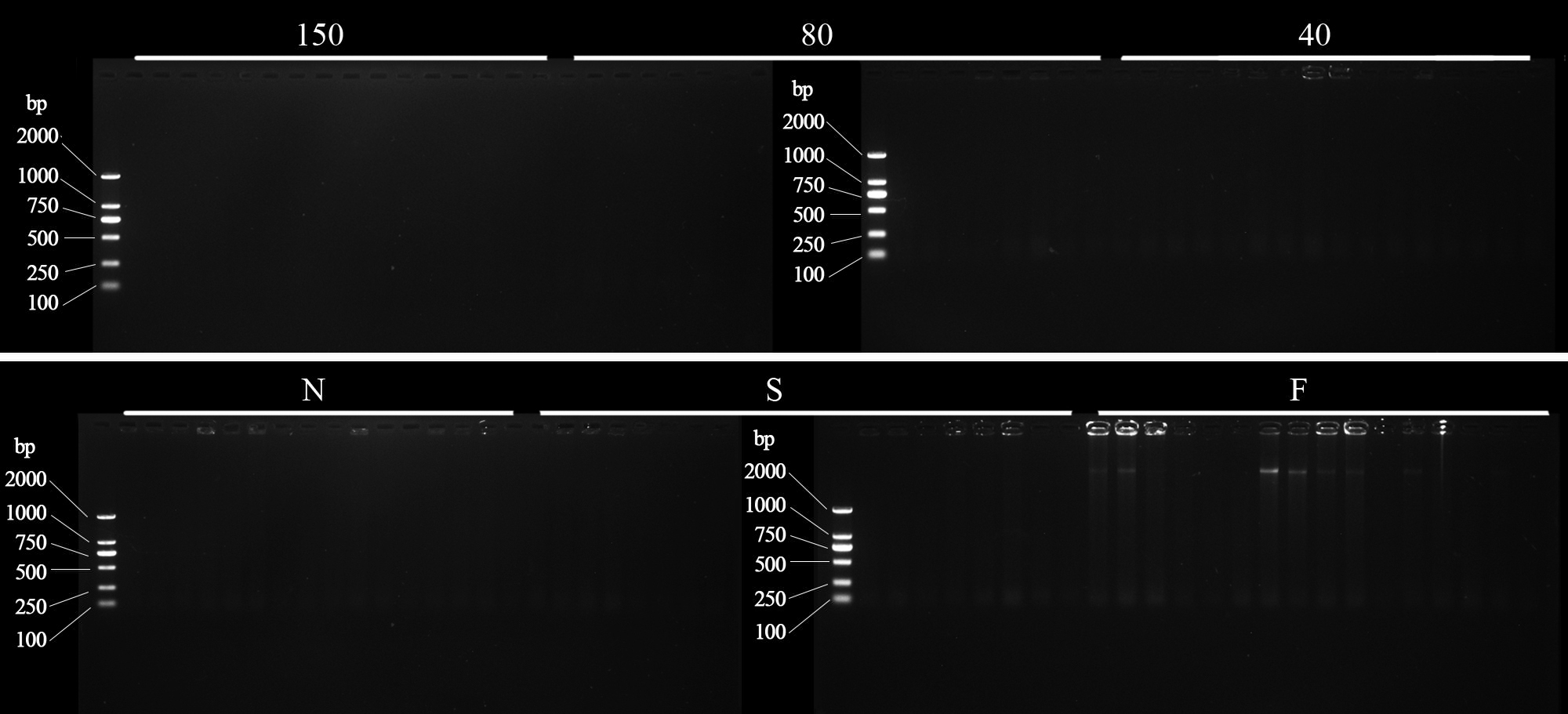

Supplement: S3 Fig — Note, 150, 150°C hot-air drying; 80, 80°C hot-air drying; 40, 40°C hot-air drying; N, natural drying; S, silica gel drying. (TIF) [file pone.0277778.s005.tif]

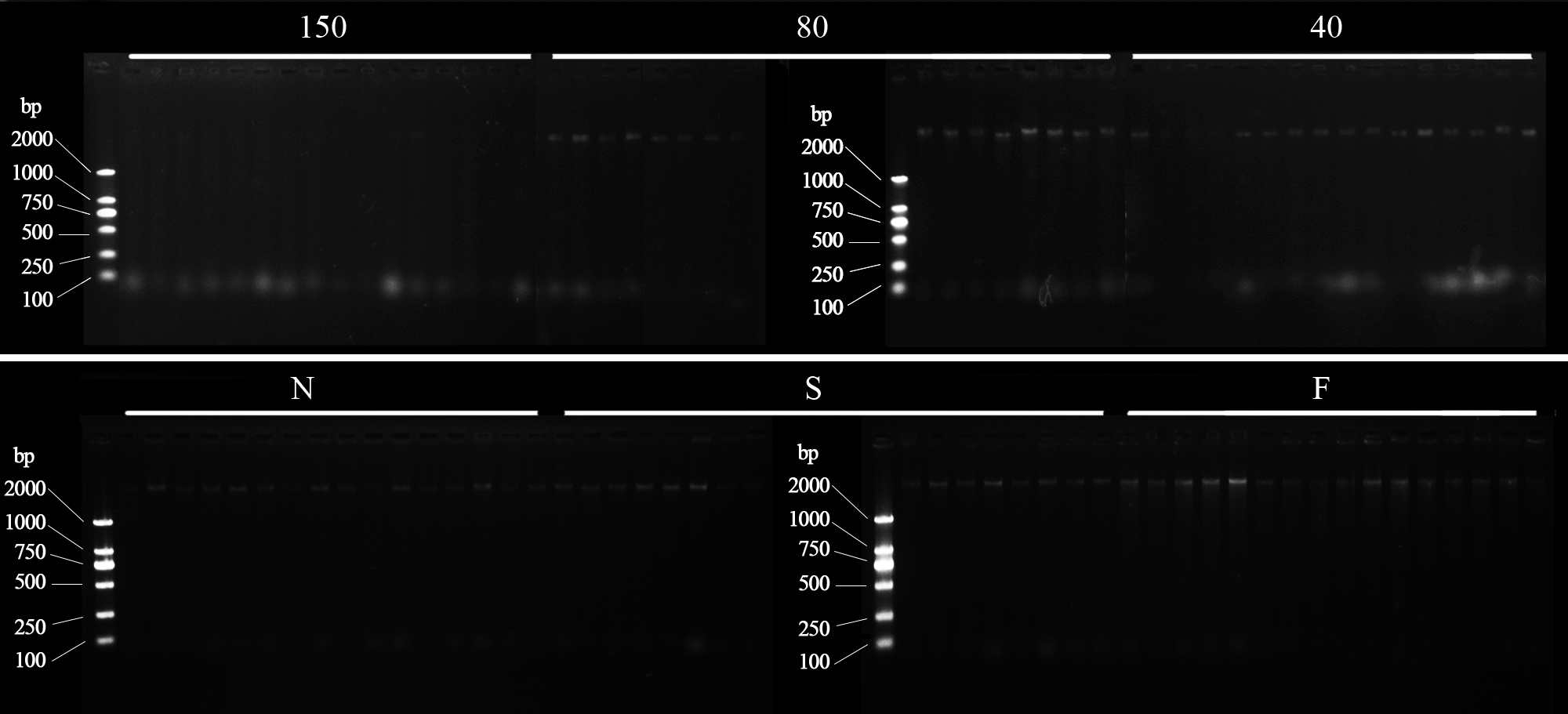

Supplement: S4 Fig — Note, 150, 150°C hot-air drying; 80, 80°C hot-air drying; 40, 40°C hot-air drying; N, natural drying; S, silica gel drying. (TIF) [file pone.0277778.s006.tif]

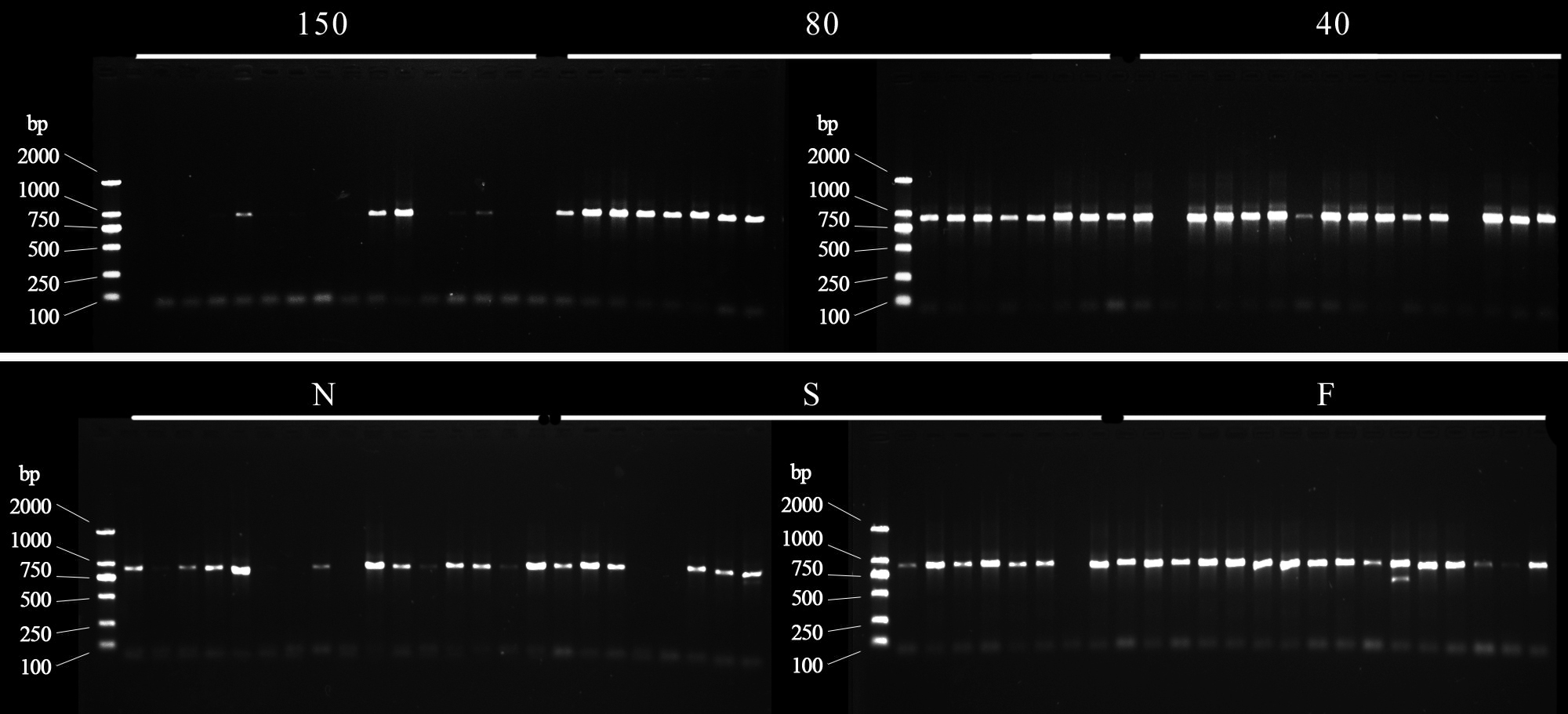

Supplement: S5 Fig — Note, 150, 150°C hot-air drying; 80, 80°C hot-air drying; 40, 40°C hot-air drying; N, natural drying; S, silica gel drying. (TIF) [file pone.0277778.s007.tif]

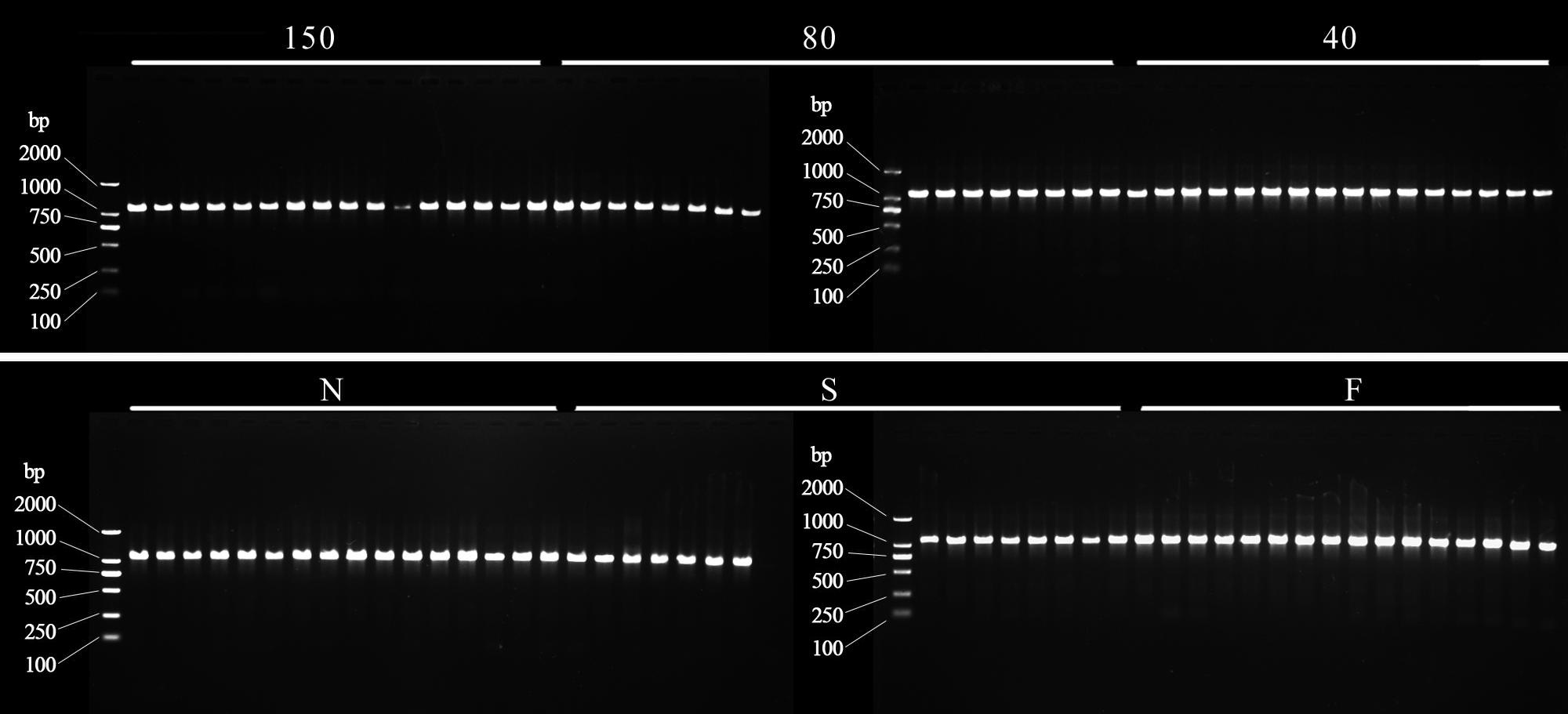

Supplement: S6 Fig — Note, 150, 150°C hot-air drying; 80, 80°C hot-air drying; 40, 40°C hot-air drying; N, natural drying; S, silica gel drying. (TIF) [file pone.0277778.s008.tif]

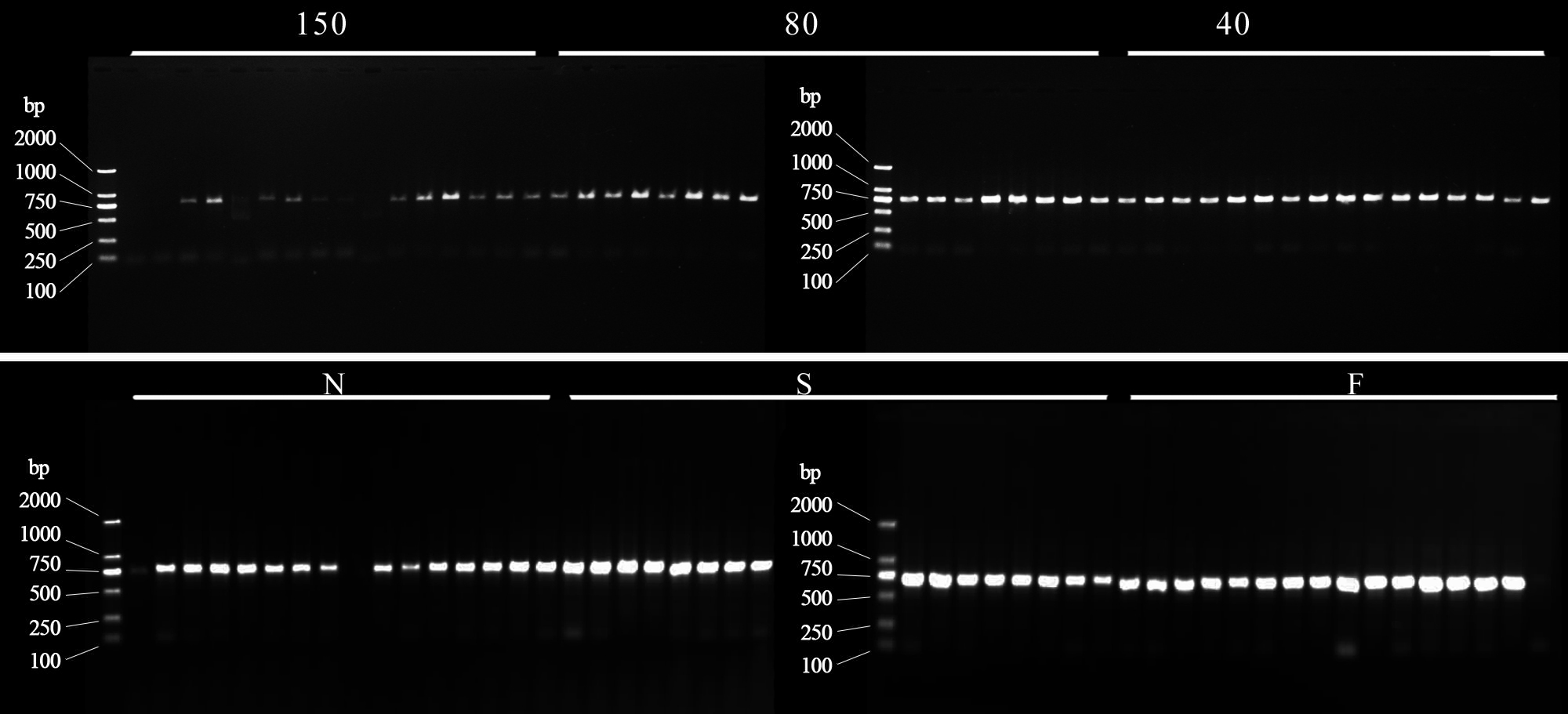

Supplement: S7 Fig — Note, 150, 150°C hot-air drying; 80, 80°C hot-air drying; 40, 40°C hot-air drying; N, natural drying; S, silica gel drying. (TIF) [file pone.0277778.s009.tif]

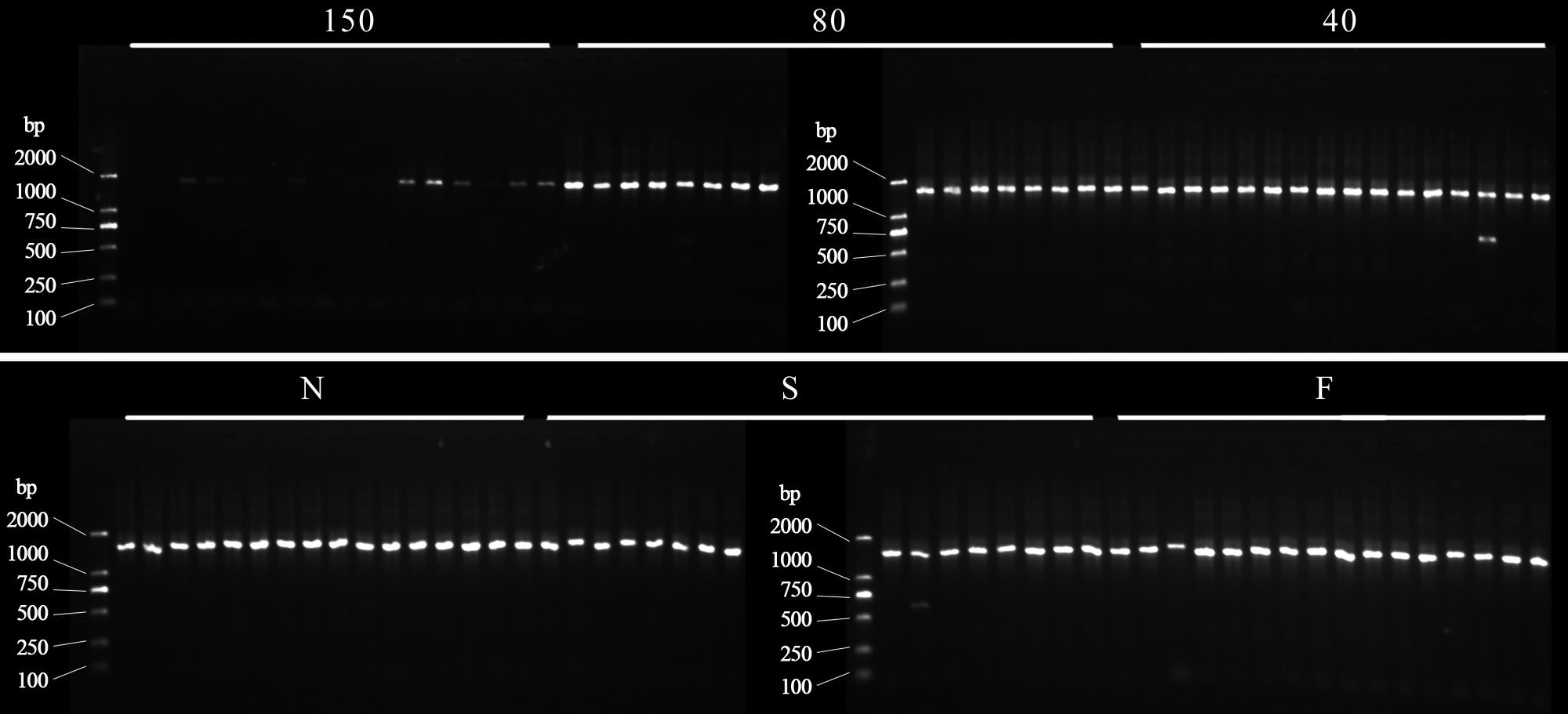

Supplement: S8 Fig — Note, 150, 150°C hot-air drying; 80, 80°C hot-air drying; 40, 40°C hot-air drying; N, natural drying; S, silica gel drying. (TIF) [file pone.0277778.s010.tif]

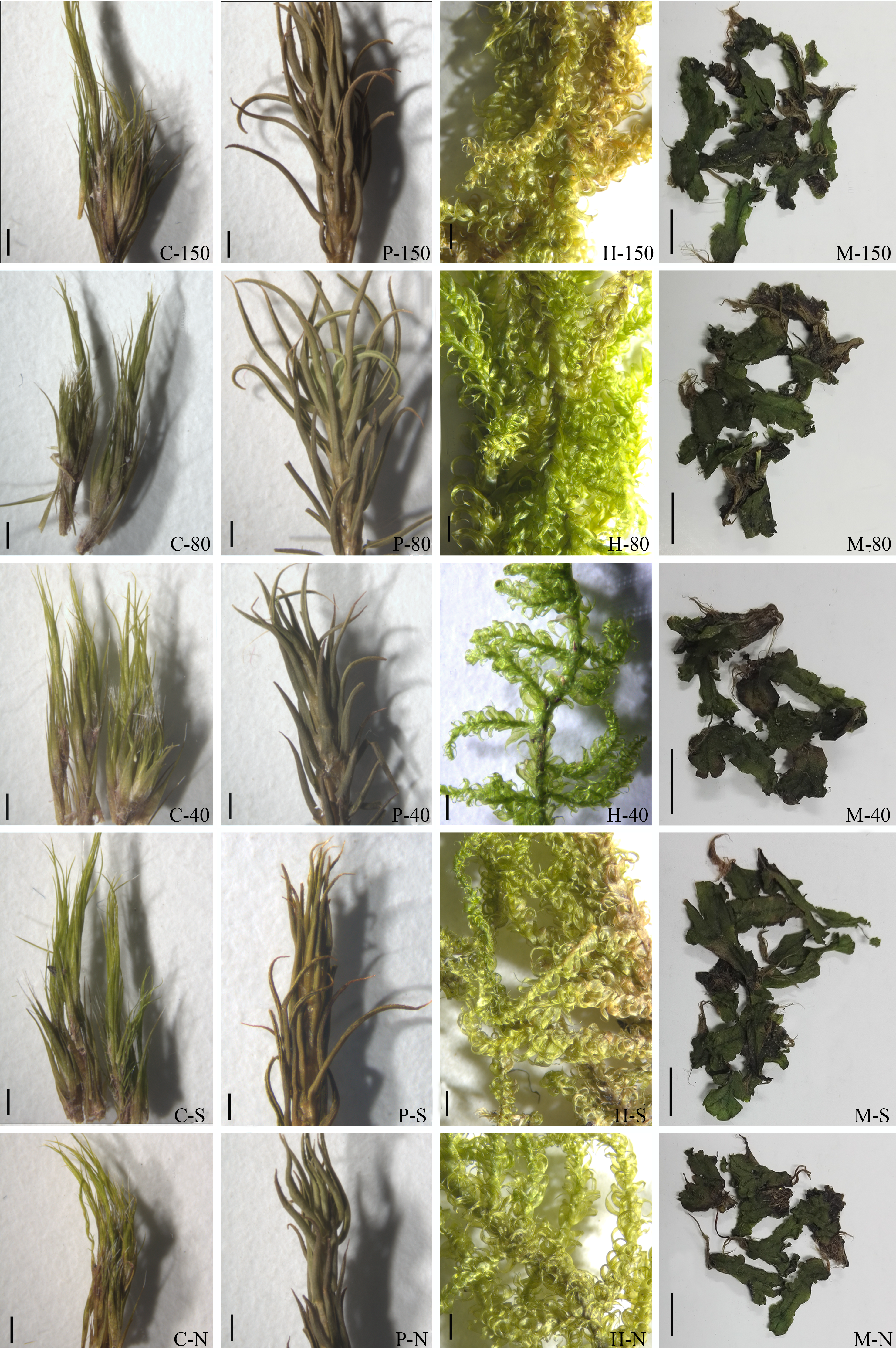

Supplement: S9 Fig — Note, C, C. schmidii; P, P. commune; H, H. calcicola; M, M. polymorpha; 150, 150°C hot-air drying; 80, 80°C hot-air drying; 40, 40°C hot-air drying; N, natural drying; S, silica gel drying. Bar scales C/P/H = 1 mm; M = 1 cm. (TIFF) [file pone.0277778.s011.tiff]

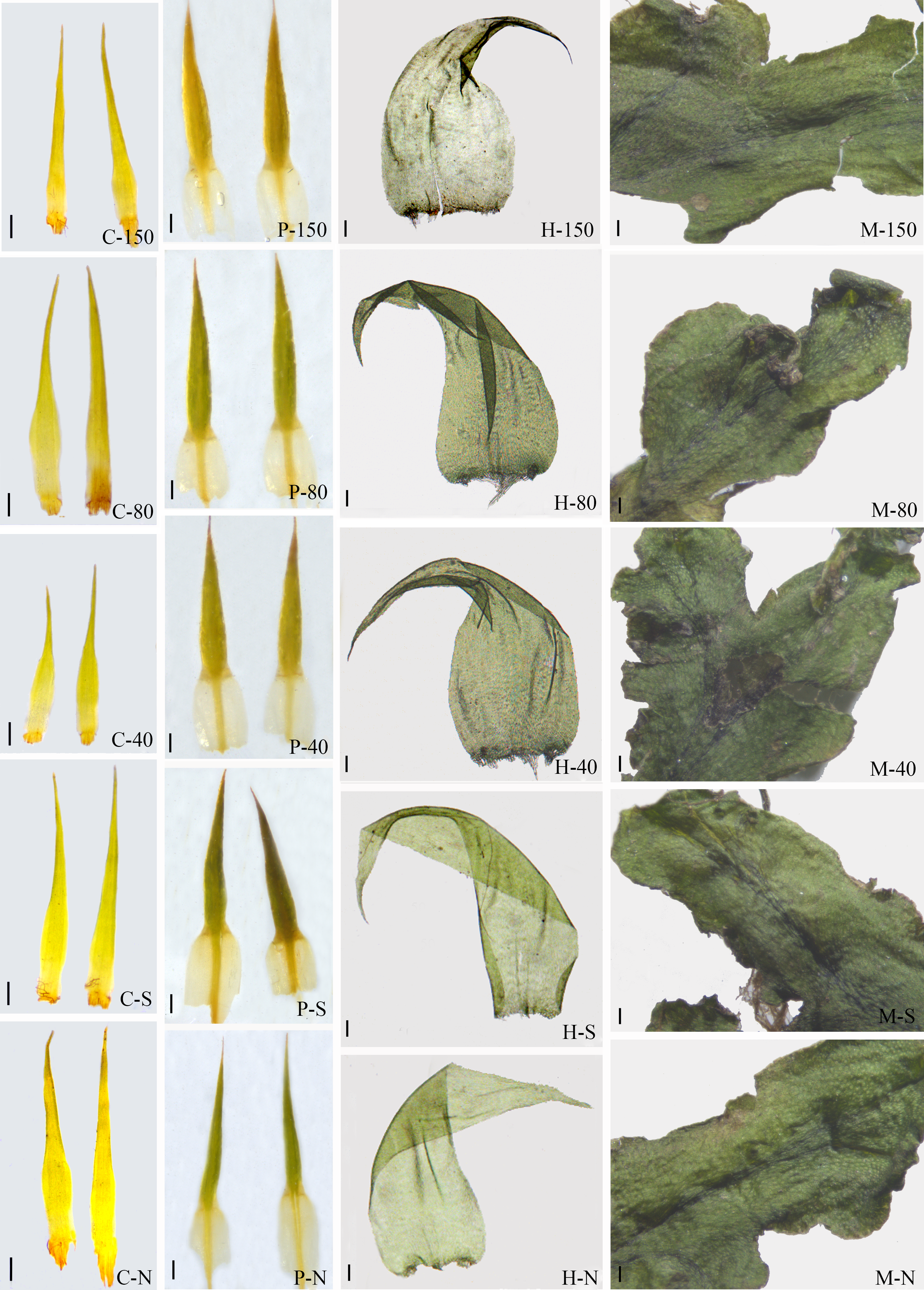

Supplement: S10 Fig — Note, C, C. schmidii; P, P. commune; H, H. calcicola; M, M. polymorpha; 150, 150°C hot-air drying; 80, 80°C hot-air drying; 40, 40°C hot-air drying; N, natural drying; S, silica gel drying. Bar scales C/P = 0.5 mm; H = 0.1 mm; M = 1 mm. (TIFF) [file pone.0277778.s012.tiff]

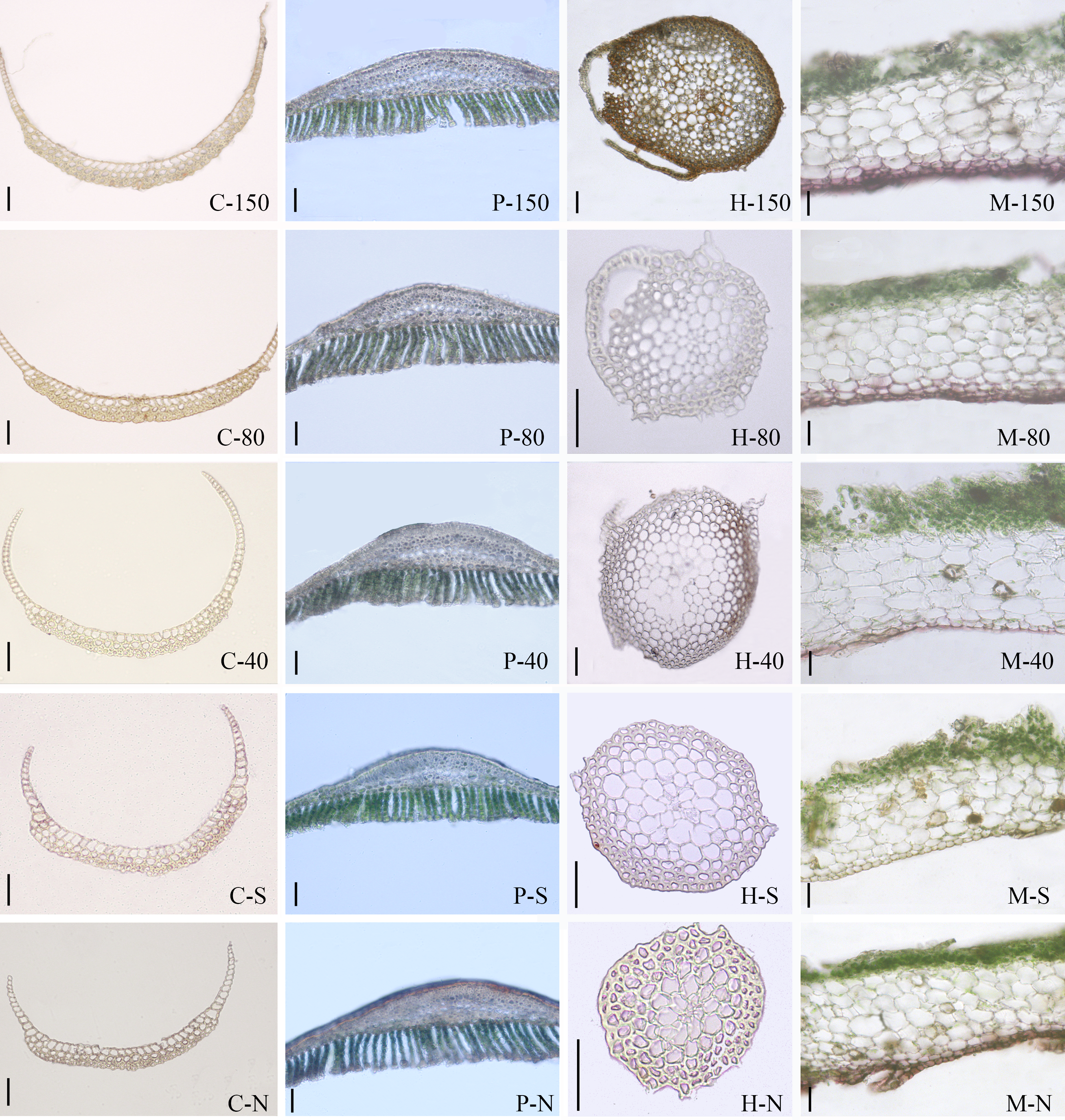

Supplement: S11 Fig — Note, C, C. schmidii; P, P. commune; H, H. calcicola; M, M. polymorpha; 150, 150°C hot-air drying; 80, 80°C hot-air drying; 40, 40°C hot-air drying; N, natural drying; S, silica gel drying. Bar scales = 50 μm. (TIFF) [file pone.0277778.s013.tiff]

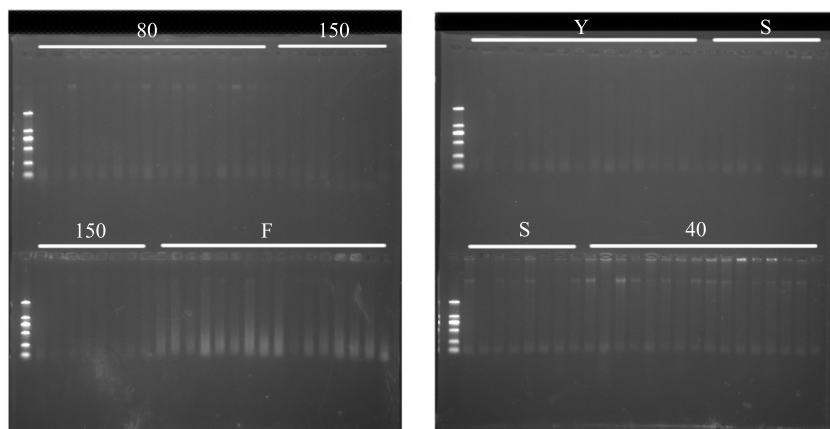

S1 Fig.

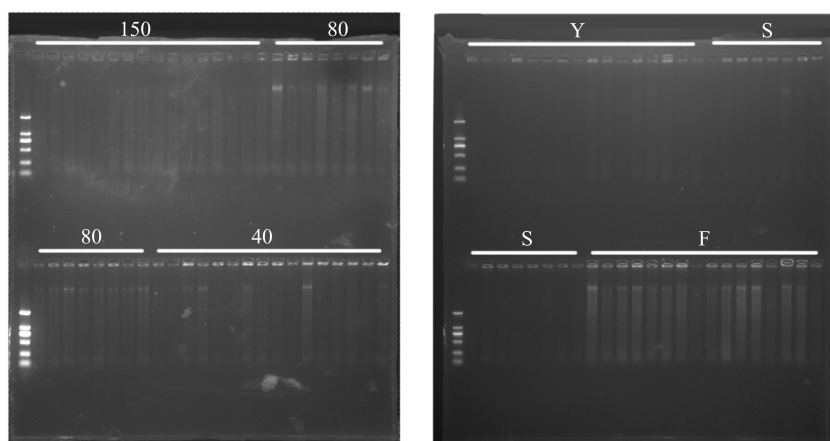

S2 Fig.

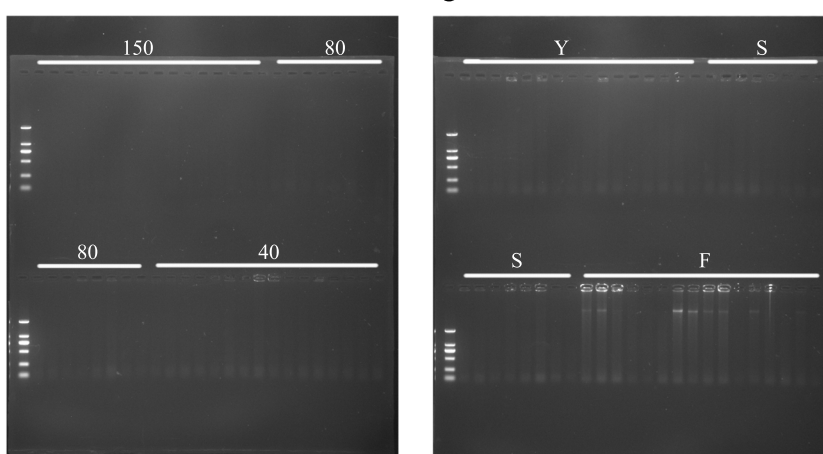

S3 Fig.

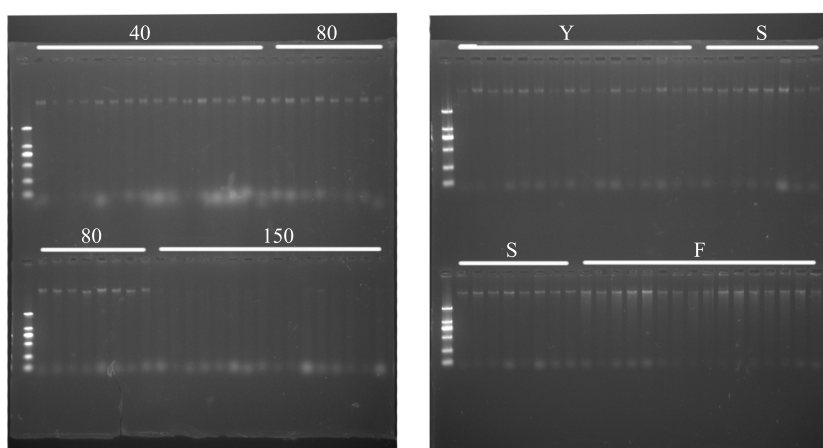

S4 Fig.

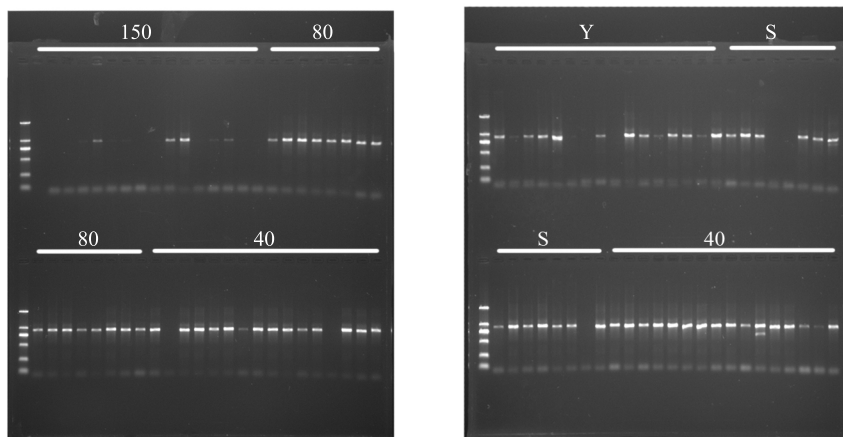

S5 Fig.

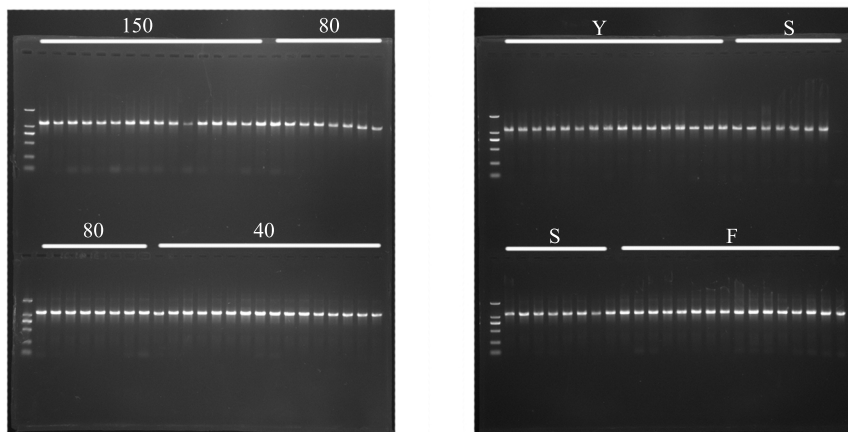

S6 Fig.

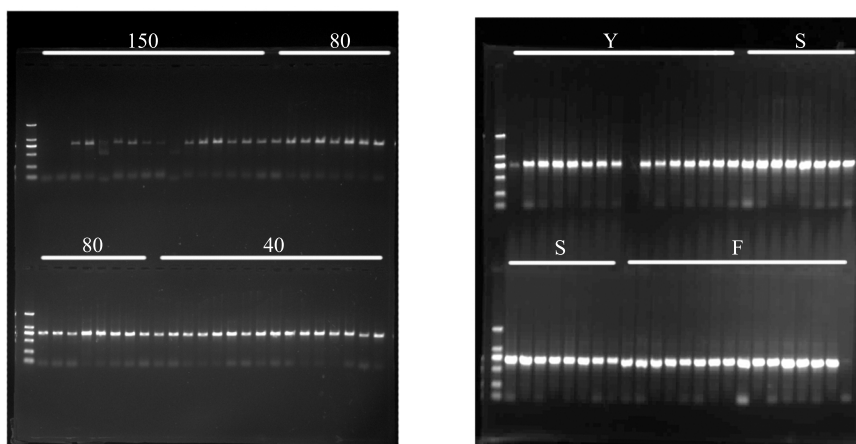

S7 Fig.

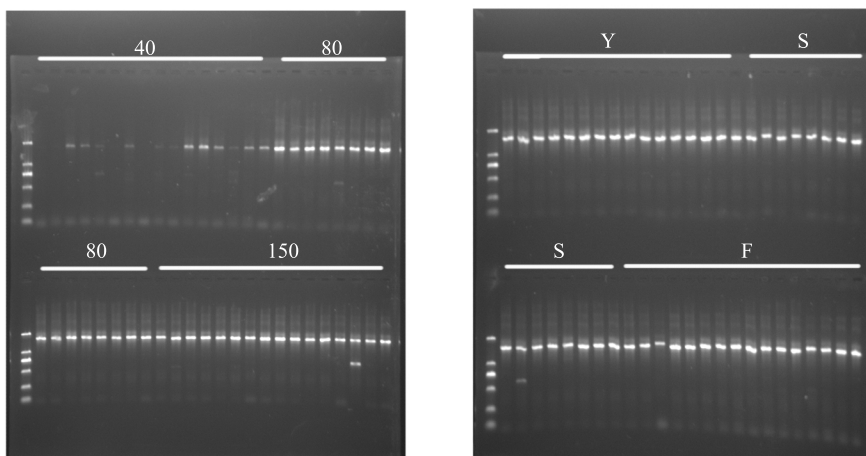

S8 Fig.

Supplement: S1 Raw images — (PDF) [file pone.0277778.s014.pdf]
